# Genetic mapping of physiological traits associated with terminal stage drought tolerance in rice

**DOI:** 10.1186/s12863-020-00883-x

**Published:** 2020-07-14

**Authors:** Saumya Ranjan Barik, Elssa Pandit, Shakti Prakash Mohanty, Deepak Kumar Nayak, Sharat Kumar Pradhan

**Affiliations:** Crop Improvement Division, ICAR-National Rice Research Institute, Cuttack, Odisha 753006 India

**Keywords:** Bulk-segregant analysis, Chlorophyll content, Proline content, Physiological traits, QTL mapping, Relative chlorophyll content, Reproductive stage drought tolerance, Rice

## Abstract

**Background:**

Drought during reproductive stage is among the main abiotic stresses responsible for drastic reduction of grain yield in rainfed rice. The genetic mechanism of reproductive stage drought tolerance is very complex. Many physiological and morphological traits are associated with this stress tolerance. Robust molecular markers are required for detection and incorporation of these correlated physiological traits into different superior genetic backgrounds. Identification of gene(s)/QTLs controlling reproductive stage drought tolerance and its deployment in rainfed rice improvement programs are very important.

**Results:**

QTLs linked to physiological traits under reproductive stage drought tolerance were detected by using 190 F_7_ recombinant inbred lines (RIL) mapping population of CR 143–2-2 and Krishnahamsa. Wide variations were observed in the estimates of ten physiological traits studied under the drought stress. The RIL population was genotyped using the bulk- segregant analysis (BSA) approach. A total of 77 SSR polymorphic markers were obtained from the parental polymorphisms survey of 401 tested primers. QTL analysis using inclusive composite interval mapping detected a total of three QTLs for the physiological traits namely relative chlorophyll content (*qRCC1.1*), chlorophyll a (*qCHLa1.1*), and proline content (*qPRO3.1*) in the studied RIL population. The QTL, *qPRO3.1* is found to be a novel one showing LOD value of 13.93 and phenotypic variance (PVE) of 78.19%. The QTL was located within the marker interval of RM22-RM517 on chromosome 3. Another novel QTL, *qRCC*1.1 was mapped on chromosome 1 at a distance of 142.8 cM and found to control relative chlorophyll content during terminal drought stress. A third novel QTL was detected in the population that controlled chlorophyll a content (*qCHLa1.1*) under the terminal stress period. The QTL was located on chromosome 1 at a distance of 81.8 cM and showed 64.5% phenotypic variation.

**Conclusions:**

The three novel QTLs, *qRCC1.1*, *qCHLa1.1* and *qPRO3.1* controlling relative chlorophyll content, chlorophyll a and proline content, respectively were identified in the mapping population derived from CR 143–2-2 and Krishnahamsa. These 3 QTLs will be useful for enhancement of terminal drought stress tolerance through marker-assisted breeding approach in rice.

## Background

Rice is an excellent gift of nature and majority of the global population consume it as staple food. Rice production and its related activities are main source of livelihood and socioeconomic security to the people in South-east Asia. Currently, about 725 million tons of paddy is being produced, worldwide, per year, from 160.8 million hectares of rice area [1]. In recent years, rice cultivation has been challenged by major production constraints mainly because of the adverse effects of climate change in India. Drought is the main yield reducing factor among the major abiotic stresses in rain-fed rice ecologies, worldwide. The frequency and occurrences of this stress is unpredictable. This stress affects rice productions in about 42 mha of rainfed rice including 8 mha upland rice in Asia [2]. In India as well, during the recent years, the rainfall pattern affects rice production to a greater extent. Therefore, high yielding varieties showing tolerance to reproductive stage drought stress need to be developed for the rainfed rice growers. Gene mapping for various traits involved during terminal drought stress tolerance and transfer of the relevant genes into superior backgrounds are needed for the improvement of rainfed rice.

The genetics of drought stress tolerance is very complex in nature and associated with several quantitative traits including various physiological and biochemical traits, involved during vegetative and reproductive stages of rice crop. Various plant traits are associated in plant growth and development during different growth stages of the crop. Majority of these traits are highly affected by drought stress, particularly during terminal stage of the crop. Even though the progress in drought breeding is slow, several traits controlled by genes/QTLs under the stress are available [3–24]. Drought stress during terminal stage is very detrimental to the crop and reduces grain yield severely [25–30]. Many physiological traits show strong correlation with drought tolerance in rice [31–37]. The highly correlated physiological traits to drought tolerance observed during terminal stage need to be mapped for deployment in the drought stress improvement in rice. Few yield QTLs are reported for enhancement of yield under drought stress under terminal stage drought [23, 38–43]. However, robust markers linked to various physiological traits involved in the stress tolerance are needed for improvement against the stress in rice.

Several research results on physiological traits responses under drought stress have already been published. Results on chlorophyll content, proline content and leaf area showed positive association with grain yield under terminal drought stress [44]. Also, the higher intensity of drought always correlated with decrease in chlorophyll content and increase in proline content in wheat plants [45]. Relative chlorophyll content (RCC) is an important physiological parameter involved during stress tolerance in rice which measures the greenness of leaves [46, 47]. Nine putative QTLs on seven different chromosomes for the trait cell-membrane stability were reported earlier under vegetative stage drought stress in rice [48]. QTL mapping study in Bala/Azucena reported 24 QTLs controlling various morphological, physiological and root related traits under drought stress, explained 4.6 to 22.3% phenotypic variance [49]. Five consistent QTLs were reported for various morphological and physiological traits linked to drought stress tolerance during terminal stage in rice [50].

Though few QTLs reports are available for drought tolerance, the need of strong molecular markers linked to various physiological traits involved during terminal stage drought tolerance are needed for molecular breeding in rice. Therefore, a RIL population comprising of 190 F_7_ lines was developed from the cross of a drought tolerant donor (CR 143–2-2) and susceptible genotype (Krishnahamsa) to map the gene(s)/QTL(s) responsible for various physiological traits conferring drought tolerance during terminal stage in rice**.**

## Results

### Estimation of physiological traits of the mapping population under terminal stage drought stress

Reproductive stage drought stress affects rice yield drastically as flowering stage is the most critical stage of rice crop. Estimates of the ten physiological traits showed significant variations in the contrasting parents recorded during both the years (Table 1). All the physiological parameters viz.*,* chlorophyll a, chlorophyll b, relative chlorophyll content, chlorophyll a + b, chlorophyll a/b, proline content, cell membrane stability, flag leaf width, biomass and per se yield were comparatively higher in the drought tolerant parent, CR143–2-2 compared to the susceptible parent except the trait, flag leaf length. Therefore, the selection of tolerant and susceptible parents for generation of mapping population may be effective.

**Table 1:** Mean value of estimates the physiological traits of contrasting parents under normal and reproductive stage drought stress situations during, 2014 and 2015

Sl. No.	phenotyping traits	Stress Condition		Normal Condition	
		CR 143–2-2	Krishnahamsa	CR 143–2-2	Krishnahamsa
1	Flag leaf length (cm)	27.96 ± 1.46	37.94 ± 1.72	28.12 ± 0.92	42.86 ± 0.89
2	Flag leaf width (cm)	1.29 ± 0.774	0.87 ± 0.052	1.5 ± 0.06	0.9 ± 0.036
3	Relative chlorophyll content	36.06 ± 1.44	27.47 ± 1.24	39.1 ± 1.56	41.6 ± 1.66
4	Biomass (g)	8.55 ± 0.941	5.25 ± 0.604	14.25 ± 1.07	19.35 ± 2.23
5	Chlorophyll a (mg/g fr. wt.)	3.45 ± 0.19	1.87 ± 0.103	5.91 ± 0.325	7.12 ± 0.391
6	Chlorophyll b (mg/g fr. wt.)	0.83 ± 0.054	0.58 ± 0.038	0.93 ± 0.047	0.97 ± 0.051
7	Chlorophyll a/b	4.16 ± 0.216	3.24 ± 0.172	6.35 ± 0.35	7.34 ± 0.403
8	Total chlorophyll (mg/g fr. wt.)	4.28 ± 0.319	2.45 ± 0.181	6.84 ± 0.383	8.09 ± 0.453
9	Cell membrane stability (%)	86.73 ± 5.64	51.14 ± 3.48	58.64 ± 3.17	46.23 ± 2.59
10	Proline content (μm/g)	160.62 ± 11.56	41.45 ± 2.94	42.34 ± 2.29	38.11 ± 2.13
11	Grain yield (g/plant)	3.71 ± 0.52	1.23 ± 0.18	9.25 ± 0.76	15.91 ± 1.29

*fr. wt.* fresh weight

Leaf area is a major trait for controlling osmosis and photosynthesis in plants. In our study, leaf length (LL) and width (LW) showed significant variations among the RILs (Table 2). LL and LW ranged from 21.5 to 46.24 cm and 0.58 to 1.5 cm, respectively. Coefficient of variations obtained for the LL and LW in the RILs were 9.6 and 9.3, respectively (Table 2). The tolerant parent, CR 143–2-2 had shorter leaf length and wider leaf width of 27.96 cm and 1.29 cm, respectively while Krishnahamsa showed longer leaf length and narrow leaf width (Table 1).

**Table 2:** Mean statistical parameters of ten physiological traits and grain yield of RILs under reproductive stage drought stress during 2014 and 2015

Traits	Mean	Range	Skewness	Kurtosis	CV(%)	LSD(5%)
LL	31.61	21.5–46.24	0.27	− 0.48	9.6	5.98
LW	0.96	0.58–1.5	0.16	−0.12	9.3	0.18
BIOM	6.78	1.21–10.73	0.79	1.41	9.3	1.24
RCC	30.61	20.14–45.95	0.54	1.6	15	8.9
CHLa	2.43	0.44–3.84	−0.01	−0.99	11	0.53
CHLb	0.55	0.07–2.39	2.13	8.38	11	0.11
CHLa+b	2.99	0.93–5.38	0.13	−0.85	9.3	0.54
CHLa/b	5.18	0.19–16.6	2.31	8.47	15	1.47
CMS	63.42	6.74–93.81	−0.78	0.4	10	12.54
PRO	74.88	6.1–228.5	1.15	0.23	17	24.84
YLD	3.75	0.82–7.68	0.79	1.38	17	0.92

Note: *LL* leaf length (cm), *LW* leaf width (cm), *BIOM* biomass (g), *RCC* relative chlorophyll content, *CHLa* chlorophyll a (mg/g fresh weight), *CHLb* chlorophyll b (mg/g fresh weight), *CHLa+b* chlorophyll a+b (mg/g fresh weight), *CHLa/b* chlorophyll a/b ratio, *CMS* cell membrane stability (%), *PRO* proline content (μm/g), *YLD* grain yield (g), *CV* coefficient of variation, LSDat 5%= least square difference

The relative chlorophyll content of tolerant the parent was higher (36.06) compared to Krishnahamsa (27.47) (Table 1). RCC estimates of RILs showed wide variation under the stress and ranged from 20.14 to 45.95 with a mean of 30.61 (Table 2). The trait showed LSD5% and coefficient of variation to be 8.9 and 15.0, respectively. A wide variation in proline content was also observed in both the parents, CR 143–2-2 (160.62 μm/g) and Krishnahamsa (41.45 μm/g). In addition, proline content in the recombinant inbred lines varied from 6.1 to 228.5 μm/g with a mean of 74.88 μm/g (Table 1). Heritability (broad-sense) was found to be high for the trait showing maximum value of 0.95. The genetic advance and genetic advance over mean exhibited higher values of 119.12 and 149.58, respectively for proline content (Table 2).

Higher estimates of chlorophyll a (CHLa) and chlorophyll b (CHLb) content were estimated from the donor parent, CR 143–2-2 based on fresh weight basis with values of 3.45 and 0.83 mg/g, respectively. The sensitive parent, Krishnahamsa, showed relatively low values of 1.87 and 0.58 mg/g, respectively (Table 1). Additionally, chlorophyll a/b (CHLa/b) and chlorophyll a + b (CHLa+b) were higher in the tolerant parent than in the sensitive one (Table 1). Higher diversity was noticed in the estimates of CHLa, CHLb, CHLa+b and CHLa/b in the RILs (Table 2). Higher PCV, GCV and heritability values were observed for CHLb with value of 55.16, 54.13 and 0.96, respectively (Table 2). Cell membrane stability (CMS) varied widely which ranged from 6.74 to 93.81% with mean value of 63.42% using the RIL population. The donor parent showed higher value of CMS (87.73%) compared to the drought sensitive parent (51.14%). Heritability (broad-sense) and genetic advance for the CMS were found to be 0.95 and 38.95, respectively. High values of phenotypic covariance (PC), environmental covariance (EC) and coefficient of variation for physiological traits were estimated from the mapping population indicated an ideal mapping population used for the physiological traits (Table 2).

### Frequency distributions

**Fig. 1:**
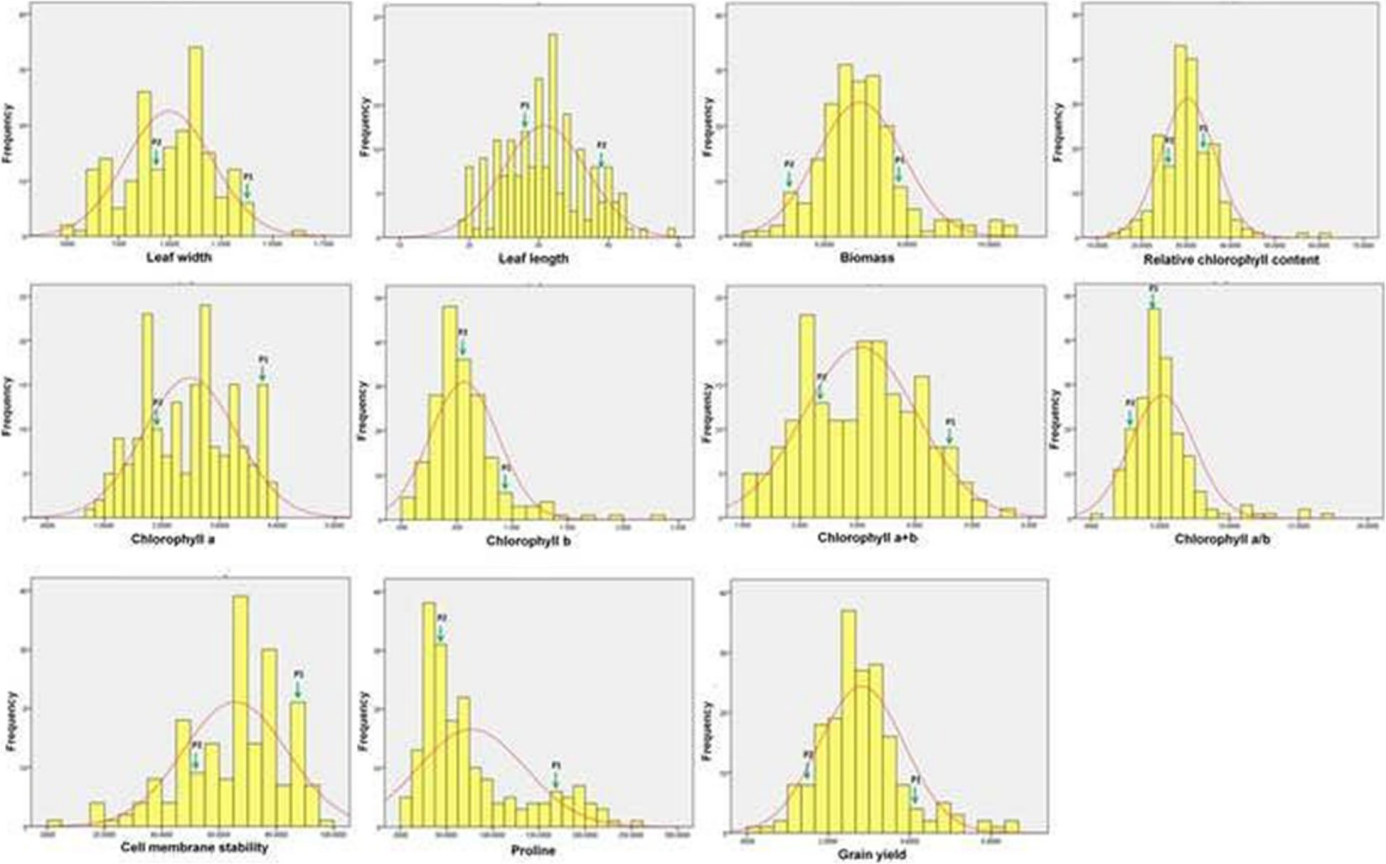
Frequency histogram and phenotypic distribution curves of ten physiological traits and grain yield generated from the RILs of CR 143–2-2 / Krishnahamsa

The distributions of recombinant inbred lines and parents based on the estimates of the ten physiological traits are shown in the Fig. 1. Both the parental lines are in the figures are depicted as P1 (tolerant parent) and P2 (susceptible parent) are placed wide apart from each other based on the studied physiological traits. Kurtosis and skewness values of the ten physiological traits for construction of a normal curve are furnished in Table 2. A positive leptokurtic skewed distribution curve was obtained for seven physiological traits viz.*,* biomass, grain yield, chlorophyll b, chlorophyll a/b, relative chlorophyll content and proline content. However, three traits viz.*,* leaf length, leaf width and chlorophyll a + b showed positive skewness estimates and negative kurtosis values. The leptokurtic distribution observed for cell membrane stability showed negative skewness and positive kurtosis. Negatively skewed platykurtic distribution was observed for the trait, chlorophyll a with both negative skewness and kurtosis value. All the studied traits except chlorophyll a and cell membrane stability showed almost a normal distribution of pattern under the stress condition (Fig.1).

### Nature of association of physiological traits and grain yield under the terminal drought stress

The correlation coefficient of the 10 physiological traits showed correlations among themselves and with grain yield (Table 3). Out of these correlation values, 26 correlations showed significant values at 0.01 and 8 were at 0.05 probability level. High positive correlations values were found for grain yield and biomass followed by chlorophyll a and chlorophyll a + b at 0.01 level of significance. Chlorophyll b and chlorophyll a/b showed a strong negative correlation (*r* = − 0.624**) at 0.01 probability level. Relative chlorophyll content, leaf length, leaf width, chlorophyll a and chlorophyll a/b showed significant positive correlation with grain yield under this stress condition (Table 3).

**Table 3:** Mean correlation coefficients of ten physiological traits and grain yield of RILs along with parents estimated under drought stress pooled over 2014 and 2015

	LL	LW	BIOM	RCC	CHLa	CHLb	CHLa+b	CHLa/b	CMS	PRO	YLD
LL	1	.404^**^	.173^*^	.111	−.023	−.120	−.055	.107	−.236^**^	−.047	.173^*^
LW	.404^**^	1	.385^**^	.044	−.085	−.191^**^	−.129	.192^**^	−.162^*^	−.193^**^	.285^**^
BIOM	.173^*^	.385^**^	1	.178^*^	.013	−.127	−.030	.187^**^	−.133	−.106	.610^**^
RCC	.111	.044	.178^*^	1	.237^**^	.152^*^	.239^**^	−.077	−.068	.042	.246^**^
CHLa	−.023	−.085	.013	.237^**^	1	.510^**^	.854^**^	−.104	.279^**^	.277^**^	.168*
CHLb	−.120	−.191^**^	−.127	.152^*^	.510^**^	1	.720^**^	−.624^**^	.247^**^	.178^*^	−.129
CHLa+b	−.055	−.129	−.030	.239^**^	.854^**^	.720^**^	1	−.276^**^	.302^**^	.279^**^	.121*
CHLa/b	.107	.192^**^	.187^**^	−.077	−.104	−.624^**^	−.276^**^	1	−.150^*^	−.003	.169^*^
CMS	−.236^**^	−.162^*^	−.133	−.068	.279^**^	.247^**^	.302^**^	−.150^*^	1	.300^**^	−.134
PRO	−.047	−.193^**^	−.106	.042	.277^**^	.178^*^	.279^**^	−.003	.300^**^	1	.205**
YLD	.173^*^	.285^**^	.610^**^	.246^**^	.168**	.121*	−.031	.169^**^	−.134	.205**	1

**Correlation is significant at 0.01 level (2-tailed)

* Correlation is significant at 0.05 level (2-tailed)

Note: *LL* leaf length (cm), *LW* leaf width (cm), *BIOM* biomass (g), *RCC* relative chlorophyll content, *CHLa* chlorophyll a (mg/g fresh weight), *CHLb* chlorophyll b (mg/g fresh weight), *CHLa+b* chlorophyll a+b (mg/g fresh weight), *CHLa/b* chlorophyll a/b ratio, *CMS* cell membrane stability (%), *PRO* proline content (μm/g), *YLD* grain yield (g)

### Mapping of physiological traits involved in reproductive stage drought tolerance

**Fig. 2:**
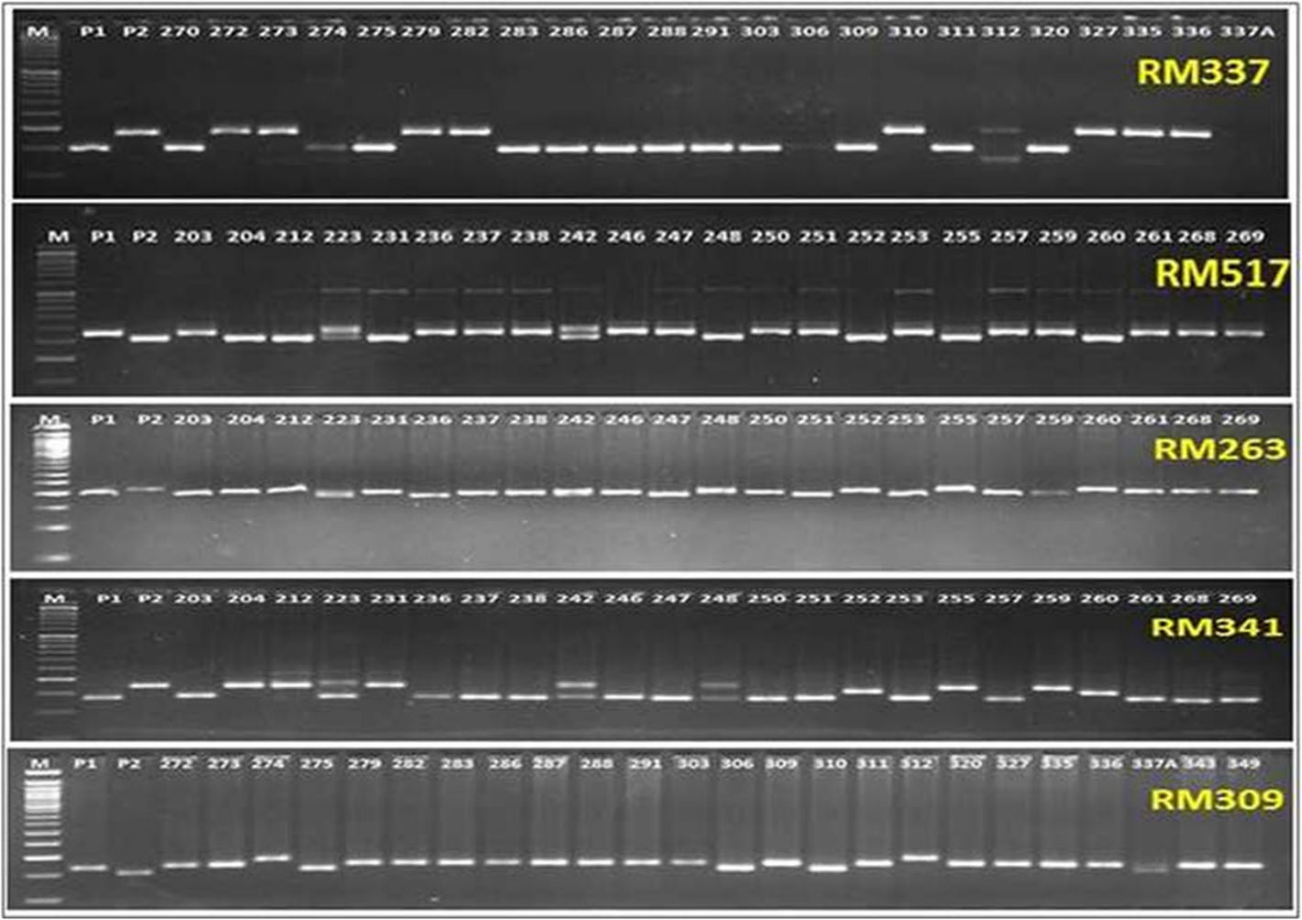
Representative electrophoregram obtained in different recombinant inbred lines using SSR markers. The numbers represent different RILs genotyped in the mapping study. Primer names are indicated in the right top corner position in each gel photos. P1: Tolerant parent, P2: Susceptible parent, M: 50 bp DNA ladder

**Fig. 3:**
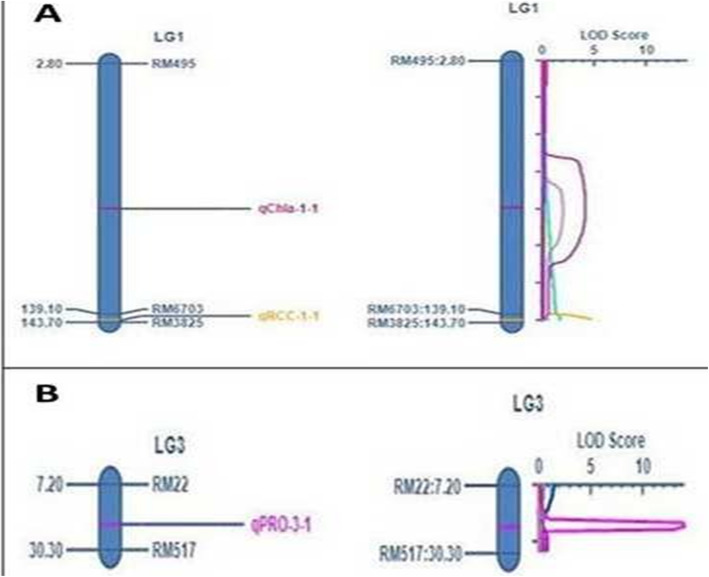
**a** QTL detected on chromosome 1 at LOD above the threshold level (violet colour represents QTL detected the Chlorophyll a and yellow colour represents QTL detected for relative chlorophyll content), **b** QTL detected on chromosome 3 at LOD above the threshold level (pink colour represents QTL detected for proline content)

In this investigation, a total of four hundred one microsatellite markers were utilized for detection of polymorphic markers between the parents (Table 4). Among the tested primers, 77 were detected to be polymorphic between the both the contrasting parents. Bulk-segregant analysis (BSA) stategy was followed by preparing two extreme inbred lines phenotypes bulks (B1: tolerant bulk and B2: susceptible bulk) and genotyped using the 77 polymorphic primers already obtained. These 77 primers were used in genotyping the RILs for mapping of the traits under the stress (Table 5; Fig. 2). ICIM (inclusive composite interval mapping) analysis revealed the presence three QTLs linked to relative chlorophyll content, proline content and chlorophyll a content under terminal drought stress situation (Table 6; Fig. 3a and b). These three QTLs showed LOD value ≥3.0 and were controlling three different physiological traits and located on two chromosomes. The QTL controlling relative chlorophyll content and chlorophyll a were found to be located on the chromosome 1 (Fig. 3). A QTL, *qPRO3.1* controlling the trait, proline content was detected on the chromosome 3 (Fig. 3). High phenotypic variance of 78.19 and LOD value of 13.93 were obtained for proline content in the mapping population (Table 6). The location of *qPRO3.1* was mapped on chromosome 3 at 21.2 cM within the marker interval of RM22 and RM517 (Table 6). A clear peak was observed for *qPRO3.1* with additive effect of − 61.5. The QTL linked to the trait was detected in both the years’ phenotypic data (2014 and 2015) using ICIM software and showed the same marker interval for the QTL, *qPRO3.1* (Table 6).

**Table 4:** Microsatellite markers obtained through the polymorphic analysis between CR143–2-2 and Krishnahamsa

Chromosome	No. of markers analyzed	Total No. and names of the parental polymorphic markers obtained		Total No. and names of the bulked polymorphic markers used	
1	50	10	RM6703, RM3825, RM488, RM259, RM5, RM12091, RM8085, RM495, RM5443, RM1003	3	RM495, RM6703, RM3825
2	50	11	RM324, RM263, RM327, RM530, RM262, RM3549, RM279, OSR17, RM250, RM 341, RM13600	3	RM327, RM341, RM263
3	42	12	RM523, RM231, RM7332, RM517, RM411, RM135, RM85, RM22, RM16030, RM15780, RM104, RM571	2	RM22, RM517
4	26	–	–	–	–
5	8	–	–	–	–
6	30	4	RM3, RM276, RM527, RM528	2	RM527, RM3
7	14	1	MGR4499	–	–
8	22	6	RM256, RM337, RM210, RM25, RM342A, RM 72	2	RM337, RM72
9	38	7	RM464, RM215, RM219, RM316, RM257, RM242, RM213	2	RM316, RM257
10	26	6	RM216, RM228, RM311, RM271, RM171, RM484	3	RM271, RM171, RM484
11	10	1	RM21	–	–
12	84	19	RM28199, RM28089, RM511, RM28166, RM1261, RM28048, RM28059, RM28064, RM28067, RM28070, RM28079, RM28082, RM28083, RM28088, RM28090, RM519, RM313, RM309, RM20A	4	RM20A, RM511, RM309, RM519
Total	401	77		21

**Table 5:** Details of polymorphic SSR markers obtained from bulk segregant analysis detected in the QTL mapping

Marker name	Chrom#	position	Forward primer	Reverse primer	Repeat motif	Annl temp
RM495	1	2.8	AATCCAAGGTGCAGAGATGG	CAACGATGACGAACACAACC	(CTG)7	55 °C
RM6703	1	139.1	CAGCAAACCAAACCAAGCC	GCGAGGAGGAGGAGAAAAAG	(TAC)12	55 °C
RM3825	1	143.7	AAAGCCCCCAAAAGCAGTAC	GTGAAACTCTGGGGTGTTCG	(GA)21	55 °C
RM327	2	72.6	CTACTCCTCTGTCCCTCCTCTC	CCAGCTAGACACAATCGAGC	(CAT)11(CTT)5	55 °C
RM341	2	94.4	CAAGAAACCTCAATCCGAGC	CTCCTCCCGATCCCAATC	(CTT)20	55 °C
RM263	2	127.5	CCCAGGCTAGCTCATGAACC	GCTACGTTTGAGCTACCACG	(CT)34	55 °C
RM22	3	7.2	GGTTTGGGAGCCCATAATCT	CTGGGCTTCTTTCACTCGTC	(GA)22	55 °C
RM517	3	30.3	GGCTTACTGGCTTCGATTTG	CGTCTCCTTTGGTTAGTGCC	(CT)15	55 °C
RM527	6	61.2	GGCTCGATCTAGAAAATCCG	TTGCACAGGTTGCGATAGAG	(GA)17	55 °C
RM3	6	92.4	ACACTGTAGCGGCCACTG	CCTCCACTGCTCCACATCTT	(GA)2GG(GA)25	55 °C
RM337	8	0.1	GTAGGAAAGGAAGGGCAGAG	CGATAGATAGCTAGATGTGGCC	(CTT)4–19-(CTT)8	55 °C
RM72	8	60.9	CCGGCGATAAAACAATGAG	GCATCGGTCCTAACTAAGGG	(TAT)5C(ATT)15	55 °C
RM316	9	1.8	CTAGTTGGGCATACGATGGC	ACGCTTATATGTTACGTCAAC	(GT)8-(TG)9 (TTTG)4 (TG)4	55 °C
RM257	9	79.7	CAGTTCCGAGCAAGAGTACTC	GGATCGGACGTGGCATATG	(CT)24	55 °C
RM271	10	59.4	TCAGATCTACAATTCCATCC	TCGGTGAGACCTAGAGAGCC	(GA)15	55 °C
RM171	10	92.8	AACGCGAGGACACGTACTTAC	ACGAGATACGTACGCCTTTG	(GATG)5	55 °C
RM484	10	102.9	TCTCCCTCCTCACCATTGTC	TGCTGCCCTCTCTCTCTCTC	(AT)9	55 °C
RM20A	12	0	ATCTTGTCCCTGCAGGTCAT	GAAACAGAGGCACATTTCATTG	(ATT)14	55 °C
RM511	12	59.8	CTTCGATCCGGT GACGAC	AACGAAAGCGAAGCTGTCTC	(GAC)7	55 °C
RM309	12	74.5	GTAGATCACGCACCTTTCTGG	AGAAGGCCTCCGGTGAAG	(GT)13	55 °C
RM519	12	94.8	AGAGAGCCCCTAAATTTCCG	AGGTACGCTCACCTGTGGAC	(AAG)8	55 °C

**Table 6:** QTL identified by inclusive composite interval mapping using the physiological traits estimated during wet season 2014, 2015 and pooled over the years

QTL detected	Year	Chrom #	Position (cM)	Left Marker	Right Marker	LOD	PVE (%)	Additive effect
*qRCC*1.1	Wet season,2014	1	142.8	RM6703	RM3825	4.77	12.43	−2.35
	Wet season,2015	1	142.8	RM6703	RM3825	4.75	12.37	−2.34
	Pooled	1	142.8	RM6703	RM3825	4.76	12.47	−2.35
*qCHLa*1.1	Wet season,2014	1	80.8	RM495	RM6703	5.4	68.65	−0.65
	Wet season,2015	1	81.8	RM495	RM6703	3.29	59.93	−0.64
	Pooled	1	81.8	RM495	RM6703	4.13	64.5	−0.65
*qPRO*3.1	Wet season,2014	3	21.2	RM22	RM517	13.93	77.77	−61.48
	Wet season,2015	3	21.2	RM22	RM517	13.93	77.79	−61.51
	Pooled	3	21.2	RM22	RM517	13.93	78.19	−61.5

Note: *RCC* relative chlorophyll content, *CHLa* chlorophyll a, *PRO* proline content, *LOD* Logarithm of the Odds and *PVE* Phenotypic variance

A QTL, designated as *qCHLa1.1* was found to be linked to the trait chlorophyll ‘a’ and present on chromosome 1 at 81.8 cM in the marker interval of RM495 and RM6703 (Table 6; Fig. 3). A small contribution of − 0.65 additive effect was obtained from this QTL mapping study (Table 6). The linkage was obtained showing phenotypic variance of 64.5% and LOD value of 4.13 for the trait, chlorophyll a (Fig. 3; Table 6). The QTL was detected using using both the years’ phenotypic data (2014 and 2015) by the ICIM software and revealed the same marker interval for the QTL, *qCHLa*. In addition, *qRCC1.1* governing the trait, relative chlorophyll content was also detected from this QTL mapping study as an important QTL showing PVE% of 12.47 and LOD value of 4.76 and detected on the chromosome 1 (Table 6; Fig. 3). A clear peak was detected in map at142.8 cM position and located in the maker interval RM6703-RM3825 (Fig. 3). This linkage region was also observed by using the 2 years’ phenotypic data and revealed the same marker interval for *qRCC1.1* (Table 6). An additive effect of − 2.35 was estimated from the analysis which is contributed by the QTL (Table 6).

## Discussion

The target physiological parameters were obtained from 190 inbred and parental lines under the stress showed wide variations among the RILs and between the parents. Few physiological traits showed strong correlations among themselves under the terminal drought stress condition. The frequency distribution curves were continuous for the studied physiological traits under the stress condition (Fig. 1). Hence, the mapping population used for tagging of the genes for the targeted physiological traits will be effective. Existence of genetic variation for relative chlorophyll content in rice genotypes under drought stress condition was also reported by earlier researchers [51]. Physiological traits namely chlorophyll a and chlorophyll b are the major traits which controll photosynthesis in plants. Reduction in photosynthetic rate under drought stress condition is attributed to chlorophyll a and b content in the leaves of rice plant [52].

In this study, large variations for the traits were observed in the RILs. Chlorophyll content of rice plant is an important secondary parameter for selection of suitable genotypes under drought stress condition due to its positive correlation with grain yield [33]. In our study, physiological traits viz.*,* chlorophyll a/b and chlorophyll a showed significant positive correlation with grain yield may be useful for the QTL study. Relative chlorophyll content is an important physiological trait use to measure the greenness that enhances photosynthesis. The analysis of relative chlorophyll content (SPAD reading) showed a positive correlation of RCC under terminal drought stress [53]. In our investigation, a significant positive correlation also observed for relative chlorophyll content with grain yield under the stress (Table 3). Another important physiological trait found to be involved under the stress condition was proline content in leaves. Proline content in the leaves showed significant increase in the tolerant lines under drought stress that enhanced the plant growth and development [54]. Under this investigation, the frequency distribution of RILs showed to be normally distributed for proline content (Fig.1). A positive significant correlation values was observed for proline content and grain yield under the stress condition. Thus, the tolerant lines showed relatively better yield under the stress with increased proline content under the stress. Therefore, increased proline content under the stress may not be antagonistic to the grain yield. In addition, highly significant correlation of proline content with chlorophyll a and chlorophyll a + b was observed. Hence, proline content may be relied as a breeding parameter during selections of drought tolerant genotypes.

Among the physiological traits studied, three parameters viz.*,* relative chlorophyll content, proline content and chlorophyll a content were linked in results mapped by QTL composite interval mapping. It is known that chlorophyll content is important for the photosynthetic process, hence highly correlated with photosynthetic rate. Low photosynthetic rate under drought stress is also associated with decrease in chlorophyll content. Low chlorophyll content in leaf reduces light absorbance in the sensitive genotypes than the tolerant genotypes. The drought sensitive genotypes could be damaged due to the damage in the photosynthetic apparatus, while drought tolerant genotypes were affected much less than tolerant genotypes. Higher lipid fluidity of the thylakoid membrane and lipid/protein ratio are seen in the tolerant genotypes, and thus increases the drought tolerance [55]. QTLs for chlorophyll content *qCC-1*, *qCC-3 and qCC8 were* detected on chromosome 1, 3 and 8, respectively within the marker interval of RG541- RG101, G62-G144 and RG598-RG418B under normal field condition in a DH population [56]. In our experiment, the linkage of the trait CHLa was detected at LOD value of 4.18 exhibiting phenotypic variance of 64.5%. The QTL, *qCHLa1.1* was located in the SSR marker interval of RM495-RM6703 on chromosome 1. No other QTLs controlling drought tolerance were reported in this chromosomal region under terminal stage drought stress. Therefore, this QTL, *qCHLa1.1* is considered as a novel QTL for controlling the trait chlorophyll a content in rice.

Another effective way to measure chlorophyll content via greenness of leaves was through SPAD meter reading. The study on quantitative trait locus for relative chlorophyll content (SPAD reading) was reported earlier by researchers [53, 57, 58]. They reported the QTL in the region of RM3916-RM2431 on the chromosome 4. Another study revealed that marker interval of RM302-RM472 on chromosome 1 was responsible for the physiological parameter for degree of greenness but undernormal field condition [59]. In our study, *qRCC1.1* was detected showing a significant LOD value, PVE (%) and additive effect of 4.76, 12.47 and − 2.35, respectively for relative chlorophyll content on Chromosome 1 flanked by marker interval of RM6703-RM3825. However, there was no earlier report of QTLs in this location for relative chlorophyll content (RCC) trait underterminal stage drought stress. Thus, *qRCC1.1* on chromosome 1 is a novel QTL controlling the trait, relative chlorophyll content at terminal drought stress situation.

Proline content in the leaves under terminal drought stress exhibited wide variation in the studied recombinant inbred lines (Table 2). High proline content is a good index for selection of tolerant genotypes under terminal drought stress tolerance in rice [60]. Proline content is an adaptive response for the accumulation of proline by plant tissue in stress condition like drought, salt and water stress [61]. Under drought condition, proline accumulation was suggested as a good parameter for drought resistance in plants [62]. The results on linkage analysis detected one QTL for proline content between marker interval of RM22-RM517 at position of 21.2 cM on the chromosome 3. The QTL showed a high LOD value and PVE (%) for the trait (Table 6). However, proline content showed high significant negative correlation with protein content but exhibited a strong positive correlation with catalase activity [60]. Experiment conducted using chromosome substitution lines for drought study revealed that the line with high proline content showed better drought tolerance and reported a segment on chromosome 1 for it [51]. The QTL detected by us was located on chromosome 3. As no earlier reports are available about any locus on the chromosome 3 controlling proline content, the QTL *qPRO*3.1 identified in our study is a novel QTL that can be useful in drought tolerance improvement in rice.

We observed a significant correlation (*r* = 0.404**) value between leaf length (LL) and leaf width (LW). These two traits were reported earlier researchers as important traits for drought tolerance which controlled canopy temperature and water use efficiency in rice [63]. But, no QTL was detected by the software for these two traits possibly due to the mapping population and mapping strategy used in this study. Also, no QTL was detected by this mapping technique for cell membrane stability (CMS). But, this trait was reported earlier as an drought breeding selection trait in cereals [48]. In this study, however, a negative correlation of − 0.134 was estimated for CMS with grain yield.

## Conclusions

The nature of association of 10 studied physiological traits among themselves and with grain yield under the terminal drought stress were studied. Relative chlorophyll content, chlorophyll a and proline content showed strong asociation with grain yield under the terminal drought stress. Three QTLs viz.*, qRCC1.1, qCHLa1.1 and qPRO3.1* for relative chlorophyll content, chlorophyll a and proline content, respectively were detected which controlled tolerance under reproductive stage drought stress. These correlated traits will be useful as selection parameters in selecting desirable progenies for enhancement of terminal drought stress tolerance in rice. The QTLs and markers detected will be much useful in molecular breeding programs for enhancement of terminal stage drought tolerance in rice.

## Methods

### Plant materials

A total of 190 RILs along with susceptible (Krishnahamsa) and tolerant (CR 143–2-2) parents were taken as the experimental materials for the mapping study. The investigation was performed under the rain-out shelter, the controlled screening facility of ICAR-National Rice Research Institute (NRRI), Cuttack, Odisha during wet the seasons*,* 2014 and 2015. CR 143–2-2 is an early duration drought tolerant line developed by Institute for upland ecology used as the donor parent. The susceptible parent, Krishnahamsa (DRR Dhan 20) is an irrigated variety of Andhra Pradesh state of India. Both the contrasting parental lines for drought tolerance were obtained from NRRI, Gene Bank. The developed RIL lines at F_7_ generation were used for phenotyping of physiological traits and genotyping using microsatellite markers.

### Phenotyping of the mapping population for physiological traits under terminal drought stress condition

All the recombinant inbred and parental lines were direct sown in an alpha lattice design using two replications during August month and irrigated up to panicle initiation stage. All the RILs were sown in 6 blocks accommodating 34 entries per block providing spacing of 10 × 15 cm. Both the parents were included in the total entries. Each row contains 25 hills per each recombinant line. Ten hill samples were collected for the evaluation of each RIL line. All the recombinant lines were grouped into three groups according to their flowering duration. Drought stress was applied at the primordium initiation (PI) stage to all the RILs and the parental lines. Fertilizer dose of 40:20:20 N:P:K was used in this the phenotyping experiment. Stress period was maintained throughout the reproductive stage and for excess stress, immediate irrigation was applied to maintain the stress up to -50kPA. Ten physiological traits viz.*,* leaf length, leaf width, biomass, relative chlorophyll content, chlorophyll a, chlorophyll b, chlorophyll a/b, chlorophyll a + b, proline content, cell membrane stability and grain yield were computed under the terminal drought stress situation.

Days to 50% flowering of the parents and recombinant lines were recorded on whole plot basis. All other pre-harvest data viz.*,* leaf length, relative chlorophyll content, and leaf width were recorded at 6–9 growth stage (SES 2014). SPAD-502 m was used to evaluate the greenness due to chlorophyll content under reproductive stage drought stress. Ten hills data were collected for recording of post-harvest data. For estimation of the studied traits namely chlorophyll a, chlorophyll b, chlorophyll a + b, chlorophyll a/b, the leaf samples were collected during mid-day. Chlorophyll content was computed by adopting the standard published method [64]. For cell membrane stability and proline content estimation, the samples were collected during mid-day period at growth stage 7–8 following the established protocols [65, 66], respectively. For estimating the relative chlorophyll content, SPAD meter or chlorophyll meter was used for recording the reading [46, 47].

### Genotyping

#### DNA extraction

Leaves of 20 days old plants were collected aseptically from different pots for extraction of total genomic DNA. Extraction procedure started with the homogenization of leaf samples using liquid nitrogen in micro centrifuge tubes along with pre-warmed (65 °C) CTAB (Cetyltrimethyl ammonium bromide) extraction buffer (2% CTAB, 100 mM Tris pH 8, 1.3 M NaCl, 20 mM Ethylene diamine tetra acetate (EDTA) pH 8) followed by extraction in chloroform isoamyl alcohol, treatment in RNase and precipitating in ethanol as described in the protocol [67]. Final product of extracted DNA was checked by comparing it with λ-DNA on 1% agarose gel for the qualitative and quantitative purpose. Also, DNA isolated was measured for quantification and purity by UV visible spectrophotometer OD at 260 and 280 nm. The DNA samples were diluted uniformly to approximately 30 ng/μl and stored for use.

#### Polymerase chain reaction (PCR)

The PCR was performed in a thermal cycler (Applied-Biosystems) described in the publication [50] using the simple sequence repeat primers (Tables 4 and 5). The reaction mix for the PCR included 30 ng genomic DNA, 1 X reaction buffer (1.5 mM Tris-HCl, 50 mM KCl, 2 mM MgCl2), 10 mM dNTPs, 1 U Taq polymerase and 5 pmole each of forward and reverse primers. The final reaction mixture volume of 20 μl was performed for polymerase chain reaction (PCR). The thermal cycler settings starts with initial denaturation at 94 °C for 4 min, denaturation at 94 °C for 30 s for 35 cycles, primer annealing at 55 °C for 1 min and extension at 72 °C for 1.30 min; final extension at 72 °C for 10 min. After completion of amplification, PCR products were stored at − 20 °C and the amplified products were analyzed by electrophoresis using 3.5% agarose gel. The DNA fragments were then visualized by using ethidium bromide dye and the banding pattern was documented using gel documentation unit (Syngene GBox).

#### Bulk-segregant analysis (BSA)

This method is used to tag the presence of major QTLs linked to the trait of interest [2]. According to the phenotypic classification of the recombinant inbred line population, 10 RILs were bulked based on the extreme tolerant and susceptible phenotypes to detect the variation based on the use of polymorphic SSR markers. Using the software ICIM V4.0, the effect of QTLs and their relation with phenotypic and molecular proportion was analyzed.

#### Statistical analysis

From the physiological trait estimates of 190 RILs and their parents during wet seasons*,* 2014 and 2015, were used for the analysis of range, mean, skewness and kurtosis to determine the phenotypic distribution, main effect of RILs with the relative traits by employing SPSS v20.0 software [68]. Also the phenotypic correlation analysis and genetic advance among the RILs were performed by INDOSTAT software [69]. Analysis for environmental variance, genotypic coefficient of variance, phenotypic coefficient of variance, heritability, and F values were computed following the previous publications [70–72].

#### Linkage map and QTL analysis

Data on ten physiological traits (leaf length, leaf width, biomass, chlorophyll a, chlorophyll b, chlorophyll a + b, chlorophyll a/b, relative chlorophyll content, proline content and cell membrane stability) and grain yield of 190 recombinant inbred lines and parents were used for construction of linkage map as described in the earlier publication [50]. The linkage map was generated by employing ICIM (inclusive composite interval mapping) v4.0 software [73]. CIM analysis and additive effect were used to calculate the association of phenotypic and molecular proportions for the construction of the map. For map construction of all QTLs, a walking speed of 1.0 cM along the chromosomes, and LOD value of 3.0 was considered as threshold value along with 1000 permutation at *P* < 0.05. The naming of the QTLs were as per the standard nomenclatural guidelines published [74].

## Data Availability

The data generated or analyzed in this study are included in this article.

## Abbreviations

BSA: Bulk-segregant analysis
CTAB: Cetyltrimethyl ammonium bromide
CHL: Chlorophyll
EDTA: Ethylene diamine tetra acetate
ICIM: Inclusive composite interval mapping
LOD: Logarithmic of odds
PRO: Proline
QTL: Quantitative trait loci
RCC: Relative chlorophyll content
RIL: Recombinant inbred line
